# Geographic distribution of cadmium and its interaction with the microbial community in the Longjiang River: risk evaluation after a shocking pollution accident

**DOI:** 10.1038/s41598-017-00280-y

**Published:** 2017-03-22

**Authors:** MingJiang Zhang, FuKe Huang, GuangYuan Wang, XingYu Liu, JianKang Wen, XiaoSheng Zhang, YaoSi Huang, Yu Xia

**Affiliations:** 1 National Engineering Laboratory of Biohydrometallurgy, General Research Institute for Nonferrous Metals, No. 2 Xinjiekouwai Street, Beijing, 100088 China; 2 Institute of HeChi Scientific-Technical Information, No. 385 West Ring Road of HeChi City, GuangXi Zhuang Autonomous Region, 547000 China

## Abstract

A shocking Longjiang River cadmium pollution accident occurred in 2012, the effects of which on microbial communities remain unclear. Alkaline precipitation technology was applied for remediation, but concerns rose about the stability of this technology. To understand the geographic distribution of cadmium and its correlation with microbes, in this study, 39 water samples and 39 sludge samples from this river and 2 soil samples from the nearby farmland were collected for chemical and microbial analyses. The Cd concentrations of all water samples were lower than 0.005 mg/L and reached the quality standards for Chinese surface water. A ranking of sludge samples based on Cd contents showed sewage outfall > dosing sites > farmland, all of which were higher than the quality standard for soil. Alkaline precipitation technology was effective for Cd precipitation. Cd was unstable; it was constantly dissolving and being released from the sludge. The Cd content of each phase was mainly influenced by the total Cd content. Over 40,000 effective sequences were detected in each sample, and a total of 59,833 OTUs and 1,273 genera were found using Illumina MiSeq sequencing. Two phyla and 39 genera were notably positively correlated with the Cd distribution, while the cases of 10 phyla and 6 genera were the opposite.

## Introduction

Cadmium (Cd) is considered as a non-essential, highly toxic metal ion^1^ in humans. A well-known example of Cd poisoning is itai-itai disease, which is caused by Cd pollution from mining waste^2^. Cd is ranked among the top ten priority hazardous substances by the Agency for Toxic Substances and Disease Registry (ATSDR) and classified as a Group 1 carcinogen by the International Agency for Research on Cancer (IARC). Cd is associated with various cancers, such as prostate, kidney, pancreas, and lung cancers^3^. Studies have indicated that chronic exposure to Cd, even at low levels, is associated with increased risk for disease, including cancer^4^. Therefore, Cd pollution not only causes serious harm to the surrounding environment but also threatens the health of humans, animals and plants^5, 6^.

In Guangxi Zhuang Autonomous Region of China, nonferrous metal industries are mainly concentrated in the Longjiang River basin, where the major grain producing area also occurs. In recent years, the unregulated discharge of untreated industrial sewage from nonferrous metal mining and enterprises has polluted the Longjiang River and posed serious threats to grain production. In 2012, there was a serious Cd pollution accident in the Longjiang River. The Cd content of the water exceeded 80 times the environment standard. This heavy pollution adversely affected the surrounding agriculture, aquaculture and residents’ livelihoods. The local government adopted alkaline chemical precipitation technology (delivered caustic soda, lime, sodium sulphide and polymeric aluminium chloride to sewage) to treat the water of the Longjiang River. Thus, Cd was precipitated in the river sludge.

Heavy metals in sludge are present in five fractions, which from the unstable fraction to the stable fraction are the exchangeable fraction, acid leachable fraction (carbonate and sulphide bound), reducible fraction (Fe and Mn oxide bound), oxidizable fraction (organic matter bound), and residual fraction (lattice bound)^7^. All fractions (except the residual fraction) can become available to biota under changing environmental conditions^8^. Cd also exists in different fractions in different environments, e.g., a significant proportion of the Cd was bound to the exchangeable and carbonate fractions in a slag disposal area^9^, the acid-soluble fraction (carbonates) and Fe oxide bound in a former mining area^10^, the carbonate (Na2EDTA) fraction in sewage sludge-amended soils^11^, the redox-sensitive fractions in wet and anoxic sediment^12^, and the residual fraction in agricultural soils^13^ and sediments of a Mexican reservoir^14^. The stability of Cd in different environments differed with its existence form. Therefore, the geographic distribution, existence form and stability of the precipitated Cd in the Longjiang River are in need of research.

Microorganisms are sensitive to heavy metals^15^, and contamination by heavy metals has a significant influence on changes in bacterial community structure^16^, microbial biomass^17^ and microbial diversity^18^. Cd is one of the heavy metals with serious environmental impacts that could also lead to significant changes in bacterial composition^19, 20^. Because of the existence of Cd, enzymatic activities and microbial biomass decrease, and some microorganisms even disappear^21, 22^. Meanwhile, it was reported that *Stenotrophomonas acidaminiphila*, *Pseudomonas aeruginosa* and *Delftia tsuruhatensis* have stronger resistance to Cd^23^. *Pseudomonas putida* and *Bacillus subtilis* are very prone to Cd accumulation^24, 25^. Some sulfate-reducing bacteria, such as *Alishewanella* sp. WH16-1, have the ability to precipitate and solidify Cd^26–28^. The effects of metal pollution on microbial communities in aquatic ecosystems have also been reported. Metals (Fe, Ni and Zn)^29^ and Cr^30^ changed the bacterial community composition of river sediments. During 12 months of incubation in mesocosms with metal-polluted harbour mud, the percentage of Cd- and Cu-tolerant aerobic heterotrophs was the highest in sandy marine sediment^31^. The microbial communities of the Longjiang River might also have been affected and changed due to the Cd pollution accident in the Longjiang River.

High throughput sequencing technology targeting 16S rRNA genes is currently widely used in research on microbial communities. In heavy metal polluted sites, high throughput sequencing has also been widely used, for example, to determine the microbial community of the Cd-, Zn-, Ni-, and Fe-contaminated Nanfei River^29^, a Leforest site with higher Cd, Zn and Mn^32^, soil containing Cd and Zn^33^, and mining soils contaminated with Cu and Zn^34^.

In this paper, water samples, sludge samples and farmland samples were collected along the entire length of the Longjiang River. Physical and chemical indices were determined for all the samples. In addition, 16S rRNA high throughput sequencing technology was applied^23, 35, 36^. The goal of this study is to investigate the stability and geographic distribution of Cd and its effects on the bacterial community composition of the Longjiang River.

## Results

### The pH of river water and Cd distribution in the Longjiang River

**Figure 1:**
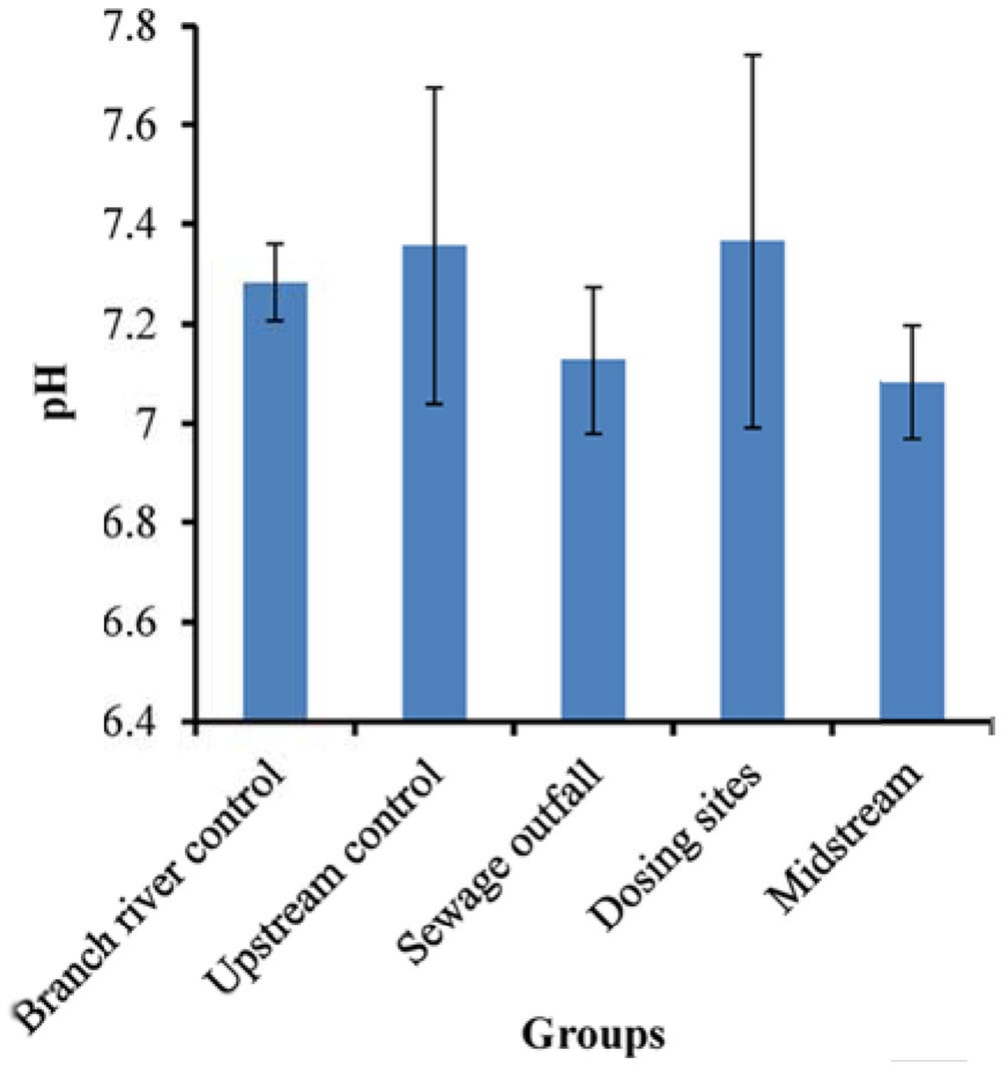
The mean pH of river water in different groups. The error bars represent standard deviation.

The pH of the river water varied from 7.0 to 7.4 in different groups (Fig. 1). Sewage outfall and midstream showed a lower pH than the control. These results implied that the effects of the unregulated discharge of untreated acid sewage several years ago remain. Dosing sites showed the highest pH. This might be due to the caustic soda and lime in the sludge of dosing sites because a weak alkaline chemical precipitation technology was used to remove Cd in 2012.

**Figure 2:**
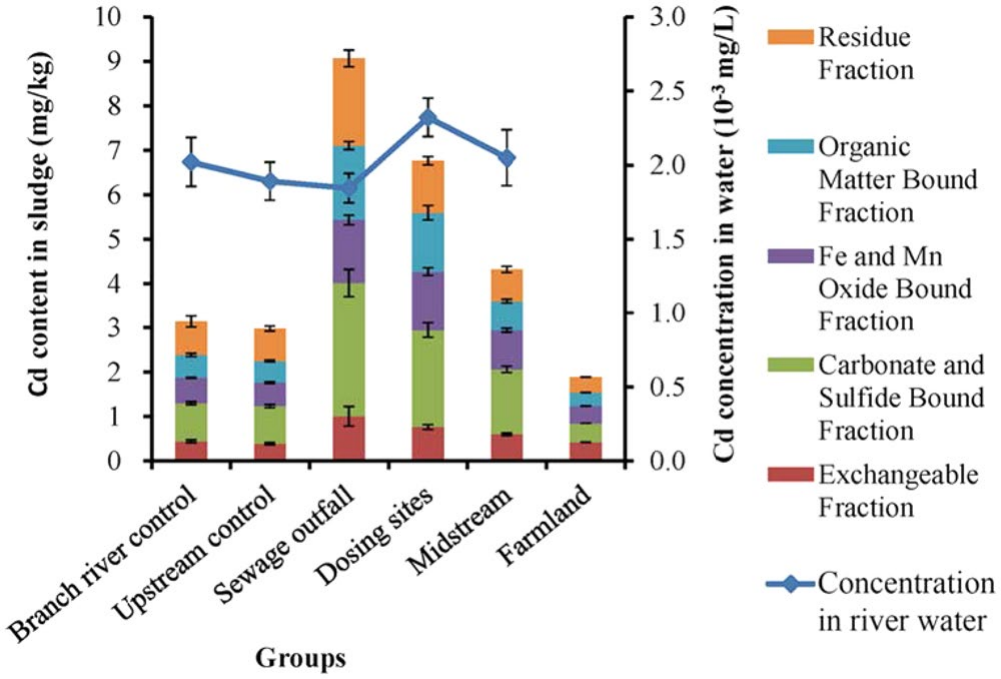
The mean Cd content of different phases in the sludge of different groups and the Cd concentration in water. The error bars represent standard deviation.

The Cd content in sludge samples and the Cd concentration in river water samples are shown in Fig. 2. In the sludge samples, sewage outfall had the highest Cd content, followed by dosing sites and midstream, and farmland had a relatively lower Cd content; the differences were significant in these groups (p < 0.05). Higher Cd content in the dosing site samples indicated that some Cd was precipitated from the river water to sludge and that the weak alkaline chemical precipitation technology used to remove Cd from river water had some effects in 2012. The amount of Cd content in the farmland samples was the lowest but still beyond the environmental quality standard for soil (GB 15618-1995). In the river water samples, dosing sites had the highest Cd concentrations (Fig. 2), which implies that Cd is unstable in dosing sites and is constantly and slowly dissolving from the sludge. The Cd concentrations of the river water in all the groups were lower than 0.005 mg/L. The quality of river water reached the second level of the national environmental quality standards of surface water (GB 3838-2002). The Cd in the sludge existed in five phases, although the total Cd content showed significant differences in different groups (p < 0.05). The composition of each phase in the different groups had no significant difference (p > 0.05). These results indicated that the Cd content of each phase was mainly influenced by the total Cd content of the sludge and had less influence from other factors.

### Microbial diversity and composition of the samples

The composition and diversity of the microbial communities in 41 samples were profiled using Illumina MiSeq platform sequencing of PCR-amplified bacterial and archaeal 16S rRNA gene fragments. After trimming and quality filtering the raw reads, 4,230,394 sequences in all and more than 40,000 sequences with the shortest length of 444 bp per sample were obtained. All of the sequences were clustered into operational taxonomic units (OTUs) using a similarity threshold of 97%, yielding a total of 59,833 OTUs. The sequences were classified into the domains of Bacteria (90.65% of the sequences and 69 phyla) and Archaea (7.97% of the sequences and 4 phyla) using the Ribosomal Database Project (RDP) classifier with a confidence threshold of 80%, with 1.38% of the sequences unclassified at the domain level. The predominant microbial phyla were Proteobacteria (30.88% ± 25.27% of the reads), Bacteroidetes (11.58% ± 9.7% of the reads), Chloroflexi (10.14% ± 8.77% of the reads), Acidobacteria (8.88% ± 6.70% of the reads) and Verrucomicrobia (7.16% ± 9.13% of the reads).

### Microbial diversity comparison between pristine and contaminated samples

Alpha-diversity analysis (Fig. S2 and Table 1) using MiSeq sequencing of the microbial 16S showed high richness and diversity, but the microbial richness and diversity in the sewage outfall and dosing sites showed a significant reduction compared to those of the control, especially in the sewage outfall samples. These results indicated that the microbial abundance was restrained and the microbial diversity reduced in the sewage outfall due to the existence of Cd, which was consistent with previous reports^35, 37^.

**Table 1:** Comparison of the estimated operational taxonomic unit (OUT) richness and diversity indices of the 16S rRNA gene libraries for clustering at 97% identity as obtained from the pyrosequencing analysis.

	Chao1	observed_species	PD_whole_tree	Shannon
Branch river control	18169 ± 686	9123 ± 501	786 ± 26	11.52 ± 0.19
Upstream control	18016 ± 435	8667 ± 177	755 ± 23	11.42 ± 0.03
Sewage outfall	15627 ± 1545	7690 ± 693	678 ± 51	11.12 ± 0.15
Dosing sites	16743 ± 737	8289 ± 271	735 ± 24	11.32 ± 0.08
Midstream	17725 ± 820	8623 ± 440	754 ± 38	11.38 ± 0.19
Farmland	15691 ± 1855	8104 ± 669	723 ± 53	11.21 ± 0.25

### Differences in microbial community composition in different samples

**Figure 3:**
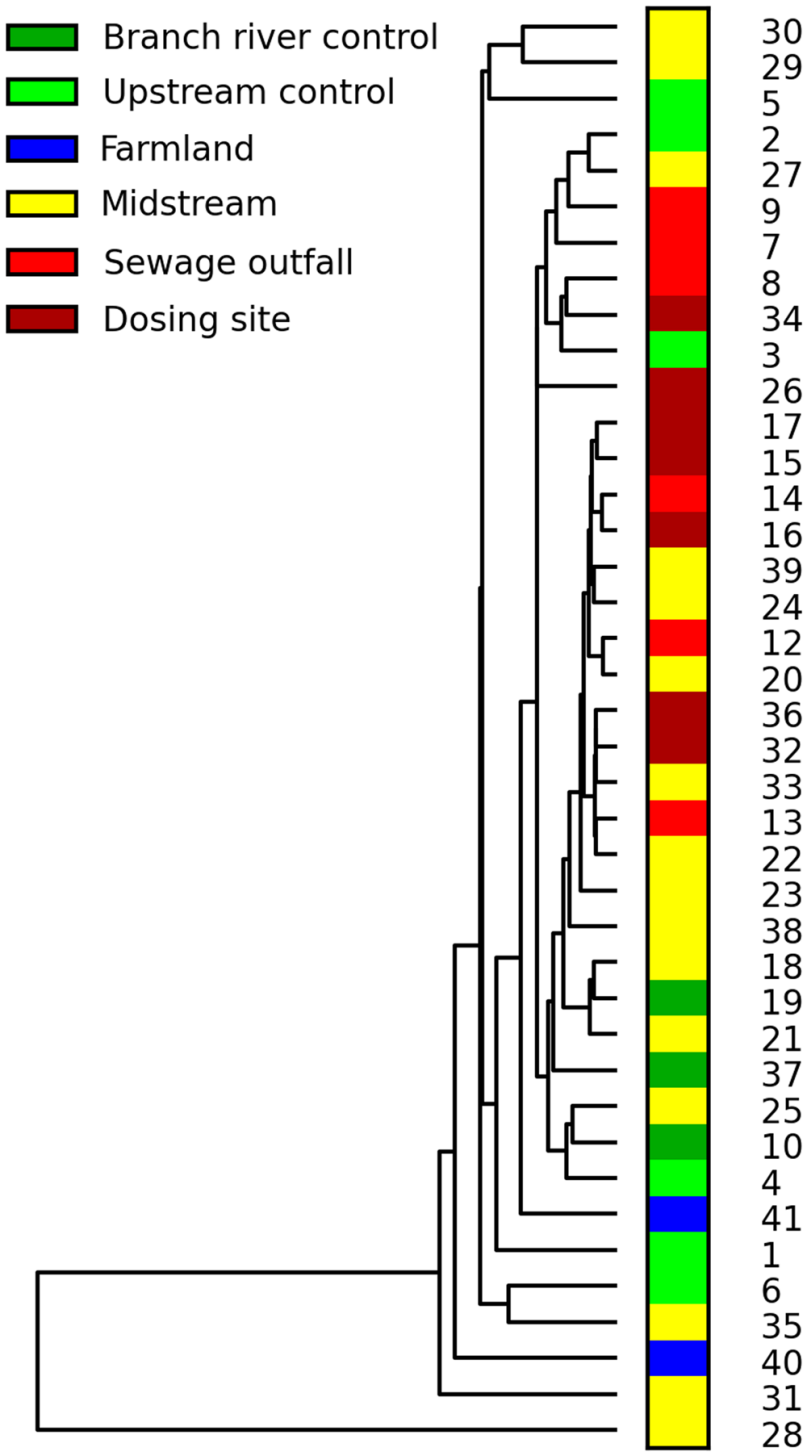
Hierarchical cluster dendrogram based on Bray–Curtis similarity obtained from all the samples.

**Figure 4:**
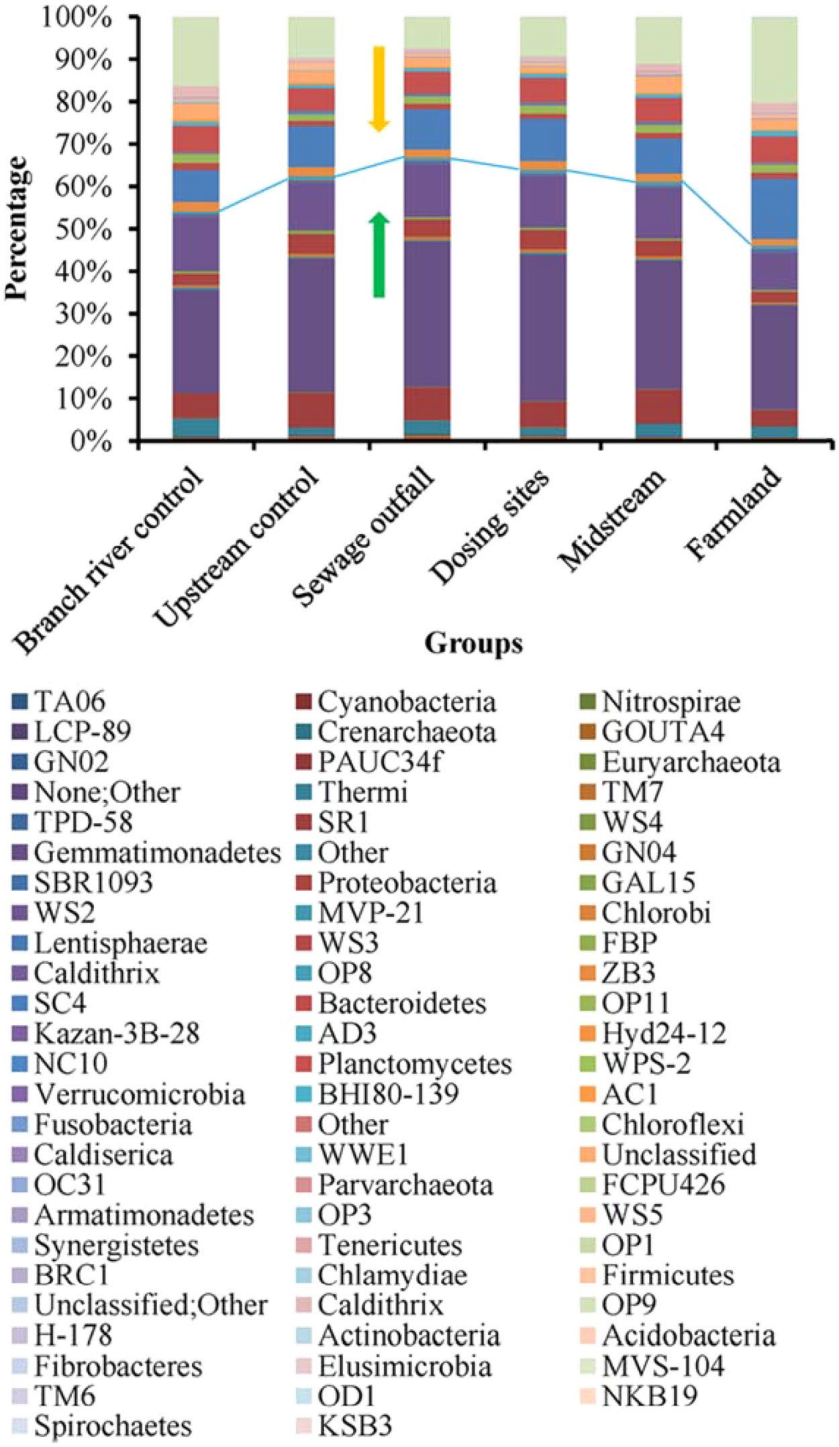
Microbial community composition of different groups (difference in relative abundance among phyla). Below the blue line are the phyla for which abundance increased in samples with high Cd content, and above the blue line are the phyla for which abundance decreased in samples with high Cd content.

The results of the analysis of community similarity among samples (Fig. 3) showed that the sewage outfall and dosing site samples mainly clustered into two large branches of the community similarity tree. Most of the pristine and contaminated samples were separated in the tree. Microbial community composition exhibited regular changes with Cd content in different groups (Fig. 4). In all 73 phyla, the relative abundances of 29 phyla increased with the increase of Cd concentration, and the largest increase (TA06) was 5.42 times. In contrast, the abundances of 44 phyla decreased, and 3 phyla could not be even detected in the samples collected from high Cd concentration sites. Among them, 2 phyla (Gemmatimonadetes and Proteobacteria) had a significantly positive correlation and 10 phyla (GOUTA4, Fibrobacteres, Kazan-3B-28, WS2, WS4, OP8, KSB3, Planctomycetes, Chlamydiae and Caldiserica) had a significantly negative correlation with Cd distribution (Table 2).

**Table 2:** Correlation between Cd content in sludge and the microbial composition at the phylum level.

Phyla	Pearson correlation	Significance
Gemmatimonadetes	0.560**	0.0003
Proteobacteria	0.368*	0.0231
GOUTA4	−0.549**	0.0004
Fibrobacteres	−0.479**	0.0024
Kazan-3B-28	−0.427**	0.0076
WS2	−0.426**	0.0077
WS4	−0.420**	0.0087
OP8	−0.381*	0.0184
KSB3	−0.372*	0.0216
Planctomycetes	−0.352*	0.0302
Chlamydiae	−0.330*	0.0428
Caldiserica	−0.326*	0.0455

*Represents significant at the level of 0.05. **Represents significant at the level of 0.01.

On the genus level, 1273 genera were detected from all the groups. The relative abundances of 533 genera increased with the increase in Cd concentration. The largest increase (*Leucobacter*) was 8 times, while the abundances of 562 genera decreased. In particular, 178 genera were not present in the sewage outfall samples. Among them, the abundances of 39 genera (such as *Pseudoxanthomonas*, *Halomonas*, and *Methanomassiliicoccus*) were positively correlated with the Cd distribution (p < 0.05), while those of 6 genera (such as *C1_B004*, *Clostridium*, and *vadinHB04*) were negatively correlated with the Cd distribution (p < 0.05) (Table S2).

### Correlations between sample properties and microbial community composition

**Figure 5:**
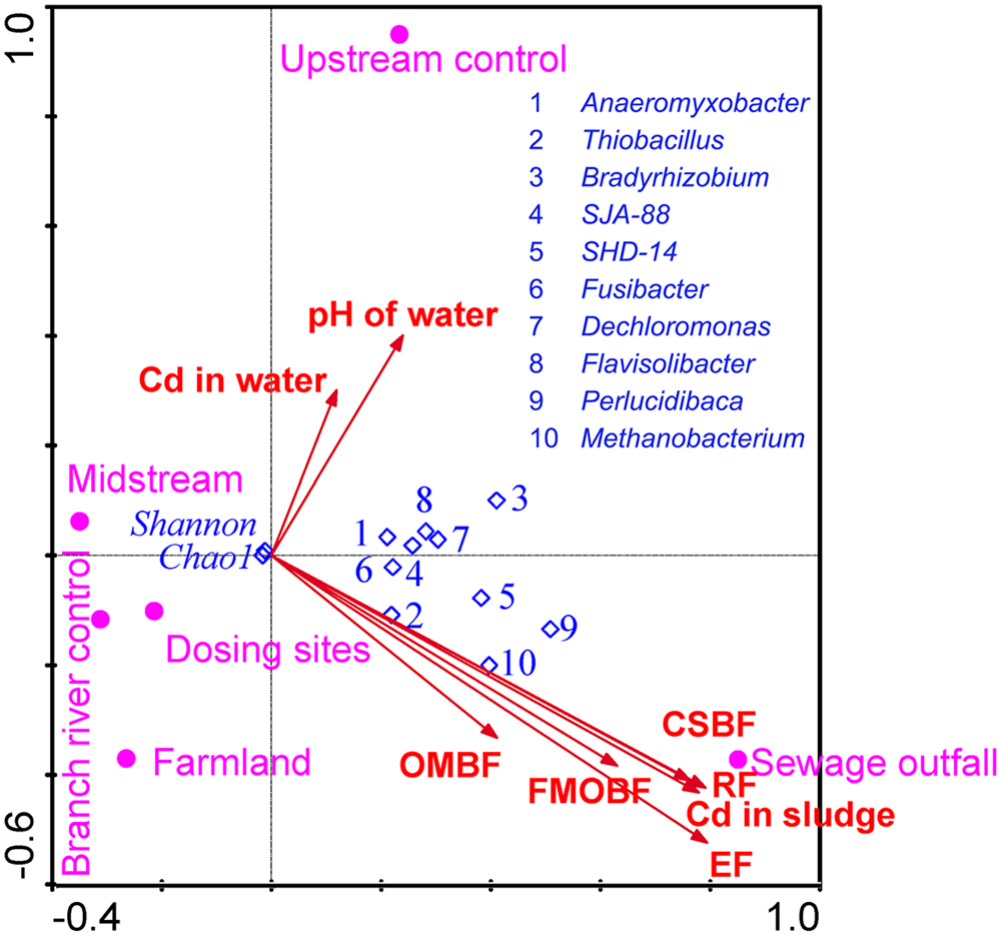
Canonical correspondence analysis. Canonical correspondence analysis triplots show the relationship between microbial composition at the genus level and sample properties. Samples are shown with solid circles, environmental parameters with solid line and solid arrows, and species, microbial richness (Chao1 index) and diversity (Shannon index) with diamonds. Exchangeable Fraction (EF), Carbonate and Sulphide Bound Fraction (CSBF), Fe and Mn Oxide Bound Fraction (FMOBF), Organic Matter Bound Fraction (OMBF), Residue Fraction (RF).

The relationships between environmental factors and microbial communities were evaluated by the canonical correspondence analysis (CCA) method (Fig. 5). All the environmental factors were distributed on the right side of the CCA plots and were positively correlated with the first axes. The microbial communities of the sewage outfall had the highest correlation with the Cd content of the sludge. The microbial communities of other groups were subjected to relatively smaller impacts from the environmental factors. *Anaeromyxobacter*, *Thiobacillus*, *Bradyrhizobium*, *SJA-88*, *SHD-14*, *Fusibacter*, *Dechloromonas*, *Flavisolibacter*, *Perlucidibaca* and *Methanobacterium* were the top 10 most abundant genera that had significantly positive correlations with the Cd content of the sludge.

Significant correlations between the Cd content in the sludge, the Cd content in different phases, microbial richness (Chao1 index) and microbial diversity (Shannon index) in the sludge samples were detected, as shown in Table 3. There were notable positive correlations (p < 0.01) between the Cd content of the five different phases in the sludge and the total Cd content in the sludge. There were no significant correlations between the Cd content of the sludge and microbial richness or microbial diversity (p > 0.05).

## Discussion

There was a positive correlation (Table 3) between the Cd content of the sludge and the Cd concentration in the river water in most of the groups (except sewage outfall). However, in sewage outfall, the highest Cd content was found in the sludge, and there was a lower Cd concentration in the water. This might be because the sewage outfall has been hardened with cement and there was less sludge in the sewage outfall. Although the highest Cd content occurred in the sewage outfall, the total amount of Cd in the sewage outfall was less than that found in the other groups. This explains the result of no significant correlation between the Cd content of the sludge and the Cd concentration of the river water (p > 0.05).

**Table 3:** Correlation between Cd content in sludge and Cd content in different phases, microbial richness (Chao1 index), and microbial diversity (Shannon index) in sludge samples.

Correlation with Cd content in sludge	Pearson correlation	Significance
Cd concentration in water	0.071	0.667
Exchangeable Fraction	0.756**	0.000
Carbonate and Sulphide Bound Fraction	0.966**	0.000
Fe and Mn Oxide Bound (Reducible) Fraction	0.755**	0.000
Organic Matter Bound (Oxidizable) Fraction	0.632**	0.000
Residue Fraction	0.821**	0.000
Chao1	−0.091	0.577
Shannon	−0.212	0.190

**Represents significant at the level of 0.01.

There were notable positive correlations (p < 0.01) between the Cd content of the five different phases in sludge and the total Cd content in the sludge. These results indicated that the Cd content of each phase in the sludge was mainly influenced by the total Cd content of the sludge rather than other factors. There were no significant correlations between the Cd content of the sludge and microbial richness or microbial diversity (p > 0.05). In particular, the farmland had the smallest correlation, which might be because there were differences in microbial taxa and microbial community structure between the river sludge samples and farmland soil samples.

There were several abundant genera in all the samples from the Longjiang River that can greatly impact the water quality of the Longjiang River and may be indicative of the water quality. For example, *Geobacter* is a genus of proteobacteria and the third largest genus of all the samples. *Geobacter* is an important metal ion-reducing bacterium that can reduce Fe(III)^38^, Pd(II)^39^ and uranium (VI)^40^. *Geobacter* can be used for the precipitation of uranium out of groundwater^41^, as a new alternative for synthesizing Pd(0) nanocatalyst, and also has potential applications for microbial metal recovery from metal-containing waste streams^39^. Additionally, *Geobacter*’s ability to consume oil-based pollutants with carbon dioxide as a waste byproduct has already been used in environmental clean-up of underground petroleum spills^42^. *Nitrospira*, from the Nitrospirae phylum, ranked the fourth in abundance in all samples. *Nitrospira* are nitrite-oxidizing bacteria that are important in marine and no marine habitats^43^. In water, *Nitrospira* takes part in the nitrification process; this is important for the biogeochemical nitrogen cycle. *Desulfobulbus*, with an abundance ranking thirty-sixth, is a sulfate-reducing bacterium, which is important for the precipitation of the heavy metal Cd in the Longjiang River.

There are some microbes that are sensitive to Cd, such as *Labrys*, *Clostridium* and *Methanocella*. *Labrys* is also sensitive to other metals (Fe^2+^, Cu^2+^, and Ag^+^)^44^. *Labrys* is an important microbe for the bioremediation of contaminants in wastewater treatment, as it has the ability to biodegrade three of the most used fluoroquinolone antibiotics worldwide: ofloxacin, norfloxacin, and ciprofloxacin^45^. The decreased abundance of *Labrys* may cause harmful effects to the environment. In contrast, the decrease of some microbes is good for the environment. For example, some species of *Clostridium* are pathogenic bacteria^46^. *Clostridium* can produce botulinum toxin in food or wounds and cause botulism^47^ and are also the causative organism of tetanus^48^. *Methanocella* is also a Cd-sensitive microbe that mainly exists in rice paddy soil and could produce methane^49^. The existence of *Methanocella* in rivers significantly contributes to methane emissions and causes global warming^50^; the abundance in the Longjiang River could reduce the production of methane.

The results of the sample similarity tree and microbial community composition indicated that the microbial community was affected by Cd. The microbial communities of sewage outfall had the highest correlation with the Cd content of sludge. This result indicated that the microbial communities of sewage outfall are subjected to the greatest impact from the Cd of sludge. There were 39 genera showing a significantly positive correlation with the sludge Cd content, some of which were beneficial for heavy metal solidification and remediation, such as *Pseudomonas*, *Desulfovibrio*, *Anaeromyxobacter*, *Leucobacter* and *Halomonas*. *Pseudomonas* contains a Cd resistance (cadR) gene^51^. It is tolerant to other heavy metals^52^ and has the ability to adsorb heavy metals^53^. In addition, *Pseudomonas* has the potential to promote plant growth, remove heavy metals from contaminated soil^54^ and immobilize heavy metals from solution^55^. Therefore, *Pseudomonas* is a new potential resource for the remediation of Cd and other heavy metals. *Desulfovibrio* is the first reported acid-tolerant gram-negative sulfate-reducing bacteria resistant to high concentrations of metals. Some *Desulfovibrio* species have bioremediation potential for the treatment of metal-containing wastewater^56^, with applications such as Hg methylation^57^ and U reduction^58^. There are two important protein types in *Desulfovibrio*. The first is orange protein (ORP, 11.8 kDa), which contains a mixed metal sulphide cluster of the type [S_2_MoS_2_CuS_2_MoS_2_]^3−^ noncovalently bound to the polypeptide chain^59^. The second is cobalt- and zinc-containing adenylate kinases (AKs)^60^. A novel type of metal-binding site for three metal ions: cobalt, zinc and iron (II) is reported to be present in AKs^61^. *Anaeromyxobacter* is an arsenate-respiring bacterium isolated from arsenic-contaminated soil that contains three distinct arsenic resistance gene clusters (ars operons)^62^. *Anaeromyxobacter* could reduce not only dissolved arsenate but also arsenate adsorbed on the soil mineral phase^63^. It might play a role in arsenic detoxification from these environments. *Leucobacter* is a chromate-resistant strain that was reported to be able to grow in a medium containing up to 300 mM K_2_CrO_4_ and showed cellular aggregation in response to chromate stress^64^. *Leucobacter* also showed strong Ni(II) removal efficiency by biosorption^65^. All these microbes have favourable functions for environment remediation and may therefore play important roles in the Longjiang River. In contrast, the increase in abundance of *Halomonas* with Cd content may be unfavourable for the environment. For example, *Halomonas* have been called “rust-eating bacteria”; they have been reported to be detected in the rusticles of the Titanic and could consume metal, causing rapid decay of the metal^66^. *Halomonas* might be unfavourable for Cd solidification because it might consume Fe of the Fe-Mn oxide bound fraction and accelerate the dissolution of Cd.

Several microbes in the samples showed significant correlations with Cd content. The relative abundances of some microbes, such as *Leucobacter*, in the sewage outfall samples were 8 times that of all samples. However, these populations accounted for a small proportion of the abundances in all samples. This might indicate small beneficial effects of Cd elimination on the microbial community. The adjustment and increase in beneficial microbes are the focus and direction of our future heavy metal pollution remediation work.

## Conclusions

In this study, the geographic distribution of cadmium contents and its correlation with the bacterial community composition in the Longjiang River were analysed.

Although the water quality of the Longjiang River met the second level national environmental quality standards of surface water, the Cd contents in the farmland samples were still beyond the environmental quality standard for soil (GB 15618-1995).

Cd was precipitated from the river water to sludge, and the weak alkaline chemical precipitation technology used to remove Cd from the river water has been effective since 2012. However, the Cd in the sludge was not stable, as it was constantly dissolving and being released from the sludge to the water. The Cd content of every phase showed significant and positive correlations with the total Cd content of the sludge. The Cd content of each phase in the sludge was mainly influenced by the total Cd content of the sludge rather than other factors.

Microbial abundance and microbial diversity were limited in the sewage with higher Cd content. The abundances of 2 phyla (Gemmatimonadetes and Proteobacteria) and 39 genera (such as *Pseudoxanthomonas*, *Halomonas*, and *Methanomassiliicoccus*) had significantly positive correlations with the Cd distribution (p < 0.05), while those of 10 phyla (GOUTA4, Fibrobacteres, Kazan-3B-28, WS2, WS4, OP8, KSB3, Planctomycetes, Chlamydiae and Caldiserica) and 6 genera (such as *C1_B004*, *Clostridium*, and *vadinHB04*) had significantly negative correlations with the Cd distribution. The taxa positively correlated with Cd content might be beneficial for Cd precipitation and remediation.

## Materials and Methods

### Sampling site information

Guangxi is located in the western region of southern China (east longitude 104°26′–112°04′, north latitude 20°54′–26°24′). This region has abundant rainfall and no obvious seasonal variations. Guangxi Zhuang Autonomous Region is rich in mineral resources and is called the “home of nonferrous metal”. The Longjiang River is located in Guangxi Zhuang Autonomous Region. The Longjiang River basin is a non-ferrous metal producing region and a grain producing region. A serious cadmium (Cd) pollution accident occurred in the Longjiang River in 2012.

### Sample collection

Sampling began at the junction of Guizhou province and Guangxi Zhuang Autonomous Region and ended at the Hexi water plant in July of 2014; the sampling area is approximately 307 km in length with sampling intervals of approximately 8 km. 39 water samples and 39 sludge samples from this river and 2 soil samples from the nearby farmland were collected, and a detailed map is provided in Fig. S1. Two 500 mL water samples and two 500 g sludge samples from each river sampling site and two 500 g soil samples from each farmland site were collected using sterile plastic bottles. The water samples were collected directly from the river surface. The sludge and soil samples were collected with a Luoyang shovel. Only the middle part of the samples in the Luoyang shovel, which represented the part approximately 5 cm–10 cm under the surface, was collected in the sterile plastic bottles. All the samples for chemical analysis were stored at 4 °C before analysis. For the microbial community analysis, the sludge samples were centrifuged at 5,000 g for 10 min, and the pellets were stored at −80 °C before DNA extraction.

### Physicochemical analysis of the samples

To estimate the amount of Cd bound to different phases in the soils and sludge, the sequential extraction procedure, consisting of a series of chemical extractions, was carried out using the following steps^7^:

#### Collection of Exchangeable Fraction

1.000 g of dry sludge sample was weighed and transferred to a 50 mL capped centrifugable bottle. 15 mL of 1 M MgCl_2_ (pH = 7.0) was added, and the sample was mechanically shaken for 2 h. Then, it was centrifuged for 15 min at 8000 rpm, and the exchangeable fraction was collected from the supernatant.

#### Collection of Carbonate and Sulphide Bound Fraction

15 mL of 1 M NaAc (pH = 5.0) was added to the residue, and it was mechanically shaken for 2 h. Then, it was centrifuged for 15 min at 8000 rpm, and the carbonate and sulphide bound fraction was collected from the supernatant.

#### Collection of Fe and Mn Oxide Bound Fraction

20 mL of 0.04 M NH_2_OH · HCl was added to the residue, leached for 5 h at 96 °C and shaken occasionally. Then, another 10 mL of 0.04 M NH_2_OH · HCl was added, the solution was centrifuged for 15 min at 8000 rpm after cooling, and then the Fe and Mn oxide bound fraction was collected from the supernatant.

#### Collection of Organic Matter Bound Fraction

3 mL (0.02 M) of HNO_3_ and 10 mL of H_2_O_2_ (30%) was added to the residue, the pH was adjusted to 2.0 with HNO_3_, and the solution was heated for 2 h at 85 °C. Then, another 3 mL of H_2_O_2_ was added (adjusted to pH = 5.0 with HNO_3_), and the residue was maintained at 85 °C for 3 h. Five millilitres of NH_4_Ac (3.2 M) was added after cooling, and the residue was diluted to 20 mL with deionized water and shaken for 1 h. After that, it was centrifuged for 15 min at 8000 rpm, and the organic matter bound fraction was collected from the supernatant.

#### Collection of Residual Fraction

5 mL of HF and 10 mL of HClO_4_ were added to the residue, which was heated until the liquid was completely evaporated, and the above steps were repeated three times. Then, 1 mL of HClO_4_ was added, and the solution was heated until the liquid was completely evaporated and white smoke fumed, following which 0.5 mL of concentrated HCl was added, and deionized water was used to adjust the solution to 25 mL.

After the sequential extraction, all the samples were filtered with super membrane filters (0.2 mm pore size, Sigma-Aldrich, MO, USA) and analysed using ICP-OES^67, 68^. The river water samples were analysed using ICP-OES directly after filtration with super membrane filters (0.2 μm pore size, Sigma-Aldrich).

### DNA extraction and sequencing

The total DNA of sludge samples was extracted using an UltraClean Soil DNA Isolation Kit (MO BIO) according to the manufacturer’s instructions, and the quality and concentration of the extracted DNA was measured using NanoVue plus. 16S rRNA gene amplifications of microbes were conducted with the 340F/805 R primer set 340F: CCTACGGGNGGCWGCAG and 805R: GACTACHVGGGTATCTAATCC, which amplifies the V4 region of the 16S rDNA gene^69^. The amplification products were confirmed by electrophoresis. High throughput sequencing targeting 16S rRNA genes was conducted on an Illumina MiSeq platform^70, 71^ by SinoGenoMax (Beijing, China). All 16S raw data can be accessed on figshare (http://dx.doi.org/10.6084/m9.figshare.1396209).

### Data processing

Paired-end reads of the original DNA fragments from high throughput sequencing were merged using FLASH^72^ and assigned to each sample according to their unique barcodes. The 16S rRNA genes were processed and analysed using the open-source software QIIME^73, 74^, and in-house Perl scripts were used to analyse alpha (within samples) and beta (among samples) diversity. First, sequence reads were filtered (fastq maxee = 0.5 and fastq trunclen = 289), replication was removed, and singletons were discarded^75^. The Chimera Slayer (CS) tool was used for chimaera detection^18^. Then, the CD-HIT package^76^ and the QIIME script “pick_de_novo_otus.py”^73^ were used to identify OTUs by making a OTU table, and sequences with ≥97% similarity were assigned to the same OTUs^77^. Representative sequences for each OTU were selected, and the RDP classifier was used to annotate taxonomic information for each representative sequence^78^. To compute the alpha diversity, the OTU table was rarefied, and two metrics were calculated: Chao1 estimates microbe abundance, and the Shannon index is used to estimate the number of unique OTUs found in each sample. Rarefaction analysis was used to quantify the representativeness of the sequencing dataset^79^. Hierarchical cluster analysis was carried out using Bray–Curtis similarity based on the abundance of all OTUs in the stats package of R^80^.

### Statistical analysis

When performing the statistical analysis, 41 samples were divided into 6 groups, which were branch river control (n = 3), upstream control (n = 6), sewage outfall (n = 6), dosing sites (n = 7), midstream (n = 16), and farmland (n = 2) (Table S1). The physicochemical indices were statistically analysed with separate one-way analyses of variance (ANOVA). The correlations (correlation between Cd content of sludge and Cd concentration of river water; the correlation between Cd content of five different phases in sludge and the total Cd content in sludge; the correlation between Cd content in sludge and microbial richness (Chao1 index); the correlation between Cd content in sludge and the alpha diversity (Shannon index) of the communities in the sludge samples; the correlation between Cd content in sludge and microbial structure) were analysed with bivariate correlation, and these statistical analysis were performed using SPSS 19.0 for Windows^81^. To evaluate the effects of environmental factors on overall functional community structures, CCA was implemented with the CANOCO 4.5 software package^82^.

## Electronic supplementary material

Geographic distribution of cadmium and its interaction with the microbial community in the Longjiang River: risk evaluation after a shocking pollution accident
