# Supplementary material for: Geographic distribution of cadmium and its interaction with the microbial community in the Longjiang River: risk evaluation after a shocking pollution accident
Source: Sci Rep. 2017 Mar 22;7:227. doi: 10.1038/s41598-017-00280-y (PMC5427973; doi:10.1038/s41598-017-00280-y)
Supplement: Supplementary file 1 — Geographic distribution of cadmium and its interaction with the microbial community in the Longjiang River: risk evaluation after a shocking pollution accident [file 41598_2017_280_MOESM1_ESM.pdf]

Geographic distribution of cadmium and its interaction with the microbial community in the Longjiang River: risk evaluation after a shocking pollution accident

MingJiang Zhang[a], FuKe Huang[b], GuangYuan Wang[a], XingYu Liu[a *], JianKang Wen[a], XiaoSheng Zhang[b], YaoSi Huang[b] and Yu Xia[a]

[a]National Engineering Laboratory of Biohydrometallurgy, General Research Institute for Nonferrous Metals, No.2 Xinjiekouwai Street, Beijing, China 100088.

[b]Institute of HeChi scientific-technical information, No. 385 West Ring Road of Hechi City, GuangXi Zhuang Autonomous Region, China, 547000

Corresponding author: *wellwoodliu@163.com, 8610-82241312

Fig. S1 Geo-visual display of sampling sites using google earth 7.1.2.2041[21]
Fig. S2 Rarefaction analysis of Chao1, observed_species, Shannon

Table S1 Samples group
Table S2 Correlation between Cd content in sludge and the percentage of all kinds of microbes in 39 sludge samples on genus level

Fig. S1 Geo-visual display of sampling sites using google earth 7.1.2.2041[21]

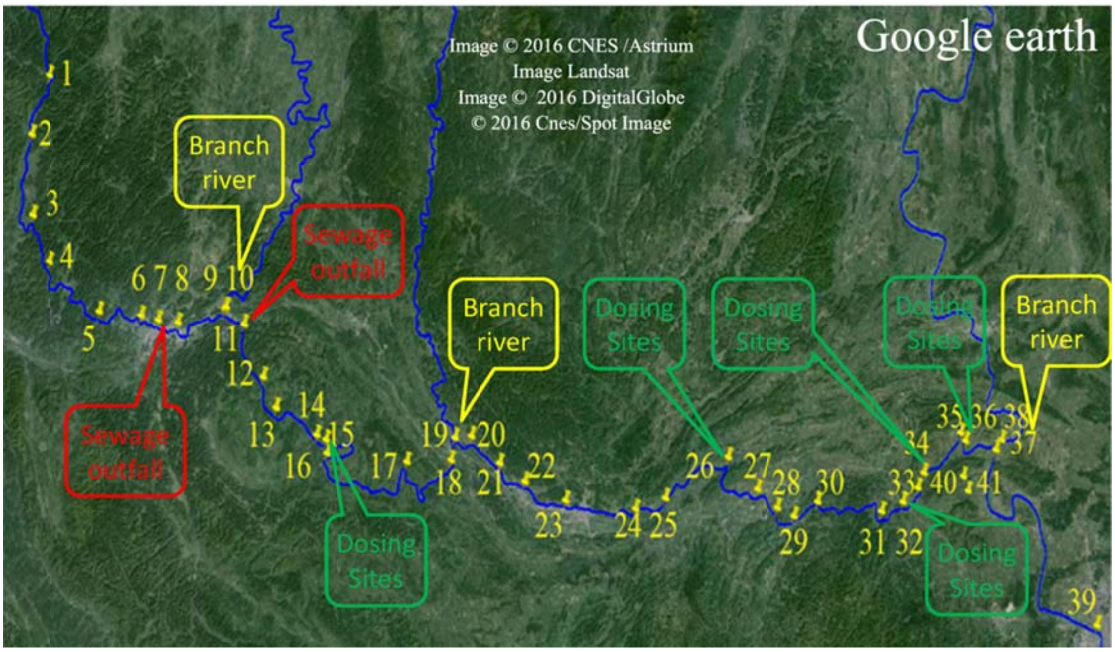

Fig. S2 Rarefaction analysis of Chao1, observed_species, Shannon

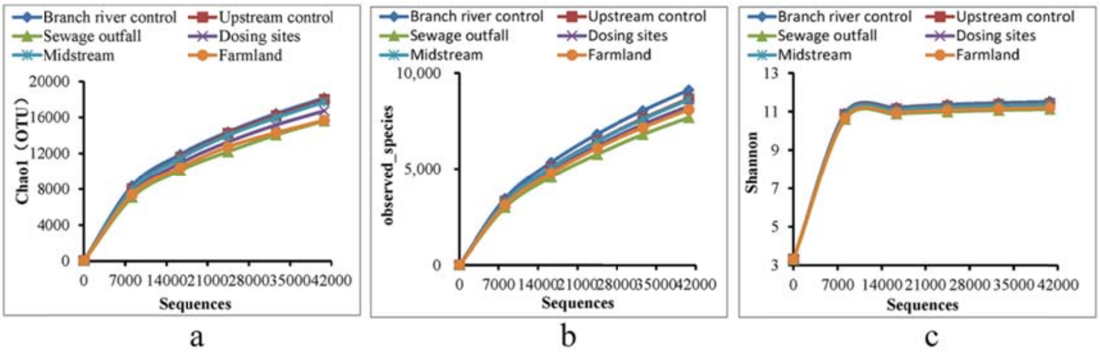

Table S1 Samples group

Groups	Samples
**Branch river control**	10, 19, 37
**Upstream control**	1, 2, 3, 4, 5, 6
**Sewage outfall**	7, 8, 9, 12, 13, 14
**Dosing sites**	15, 16, 17, 26, 32, 34, 36
**Midstream**	18, 20, 21, 22, 23, 24, 25, 27, 28, 29, 30, 31, 33, 35, 38, 39
**Farmland**	40, 41

Table S2 Correlation between Cd content in sludge and the percentage of all kinds of microbes in 39 sludge samples on genus level

Microbial kinds	Pearson correlation	Significance
k__Bacteria;p__Proteobacteria;c__Gammaproteobacteria;o__Xanthomonadales;f__Xanthomonadaceae;g__Pseudoxanthomonas	.633**	0
k__Bacteria;p__Proteobacteria;c__Gammaproteobacteria;o__Oceanospirillales;f__Halomonadaceae;g__Halomonas	.570**	0.0001
k__Archaea;p__Euryarchaeota;c__Thermoplasmata;o__E2;f__[Methanomassiliicoccaceae];g__Methanomassiliicoccus	.583**	0.0001
k__Bacteria;p__Proteobacteria;c__Betaproteobacteria;o__Burkholderiales;f__Comamonadaceae;g__Hylemonella	.562**	0.0002
k__Bacteria;p__Bacteroidetes;c__Cytophagia;o__Cytophagales;f__Cytophagaceae;g__Pontibacter	.566**	0.0002
k__Bacteria;p__Proteobacteria;c__Betaproteobacteria;o__Hydrogenophilales;f__Hydrogenophilaceae;g__Thiobacillus	.547**	0.0003
k__Bacteria;p__Proteobacteria;c__Gammaproteobacteria;o__Pseudomonadales;f__Moraxellaceae;g__Perlucidibaca	.514**	0.0008
k__Archaea;p__Euryarchaeota;c__Methanobacteria;o__Methanobacteriales;f__Methanobacteriaceae;g__Methanobrevibacter	.497**	0.0013
k__Bacteria;p__Actinobacteria;c__Actinobacteria;o__Actinomycetales;f__Microbacteriaceae;g__Leucobacter	.494**	0.0014
k__Bacteria;p__Bacteroidetes;c__Bacteroidia;o__Bacteroidales;f__Porphyromonadaceae;g__Paludibacter	.495**	0.0014
k__Bacteria;p__Chloroflexi;c__Anaerolineae;o__Anaerolineales;f__Anaerolinaceae;g__T78	.488**	0.0016
k__Bacteria;p__Proteobacteria;c__Gammaproteobacteria;o__Xanthomonadales;f__Xanthomonadaceae;g__Dokdonella	.487**	0.0017
k__Bacteria;p__Verrucomicrobia;c__[Spartobacteria];o__[Chthoniobacterales];f__[Chthoniobacteraceae];g__Ellin506	.484**	0.0018
k__Bacteria;p__Synergistetes;c__Synergistia;o__Synergistales;f__Aminiphilaceae;g__Aminiphilus	.481**	0.0019
k__Bacteria;p__Proteobacteria;c__Deltaproteobacteria;o__Desulfovibrionales;f__Desulfovibrionaceae;g__Desulfovibrio	.473**	0.0023
k__Bacteria;p__Spirochaetes;c__[Leptospirae];o__[Leptospirales];f__Sediment-4;g__SJA-88	.467**	0.0027
k__Bacteria;p__Proteobacteria;c__Deltaproteobacteria;o__Myxococcales;f__Myxococcaceae;g__Anaeromyxobacter	.460**	0.0032
k__Bacteria;p__Proteobacteria;c__Alphaproteobacteria;o__Rhodospirillales;f__Rhodospirillaceae;g__Phaeospirillum	.457**	0.0034
k__Bacteria;p__Bacteroidetes;c__Flavobacteriia;o__Flavobacteriales;f__[Weeksellaceae];g__Chryseobacterium	.454**	0.0037
k__Bacteria;p__Chloroflexi;c__Anaerolineae;o__Anaerolineales;f__Anaerolinaceae;g__C1_B004	-.444**	0.0053
k__Bacteria;p__Bacteroidetes;c__Cytophagia;o__Cytophagales;f__Cytophagaceae;g__Sporocytophaga	.402*	0.0112

k__Bacteria;p__Proteobacteria;c__Betaproteobacteria;o__Rhodocyclales;f__Rhodocyclaceae;g__Dechloromonas	.399*	0.0118
k__Archaea;p__Euryarchaeota;c__Methanobacteria;o__Methanobacteriales;f__Methanobacteriaceae;g__Methanobacterium	.396*	0.0125
k__Bacteria;p__Verrucomicrobia;c__Verrucomicrobiae;o__Verrucomicrobiales;f__Verrucomicrobiaceae;g__Verrucomicrobium	.386*	0.0151
k__Bacteria;p__Synergistetes;c__Synergistia;o__Synergistales;f__TTA_B6;g__E6	.382*	0.0163
k__Bacteria;p__Firmicutes;c__Clostridia;o__Clostridiales;f__Clostridiaceae;g__Clostridium	-.385*	0.0169
k__Bacteria;p__Proteobacteria;c__Gammaproteobacteria;o__Xanthomonadales;f__Xanthomonadaceae;g__Arenimonas	.374*	0.019
k__Bacteria;p__Bacteroidetes;c__[Saprospirae];o__[Saprospirales];f__Chitinophagaceae;g__Sediminibacterium	.372*	0.0196
k__Bacteria;p__Tenericutes;c__Mollicutes;o__Anaeroplasmatales;f__Anaeroplasmataceae;g__Asteroleplasma	.371*	0.0201
k__Bacteria;p__Gemmatimonadetes;c__Gemmatimonadetes;o__Gemmatimonadales;f__Gemmatimonadaceae;g__Gemmatimonas	.371*	0.0202
k__Bacteria;p__Firmicutes;c__Clostridia;o__Clostridiales;f__Veillonellaceae;g__vadinHB04	-.375*	0.0205
k__Bacteria;p__Firmicutes;c__Bacilli;o__Turicibacterales;f__Turicibacteraceae;g__Turicibacter	.369*	0.0207
k__Bacteria;p__Proteobacteria;c__Alphaproteobacteria;o__Rhizobiales;f__Rhodobiaceae;g__Afifella	.368*	0.0211
k__Bacteria;p__Bacteroidetes;c__Cytophagia;o__Cytophagales;f__Cytophagaceae;g__Adhaeribacter	.366*	0.0219
k__Bacteria;p__Proteobacteria;c__Betaproteobacteria;o__Burkholderiales;f__Comamonadaceae;g__Paucibacter	.353*	0.0276
k__Bacteria;p__Proteobacteria;c__Gammaproteobacteria;o__Pseudomonadales;f__Pseudomonadaceae;g__Pseudomonas	.352*	0.028
k__Bacteria;p__Firmicutes;c__Clostridia;o__Clostridiales;f__Veillonellaceae;g__G07	.350*	0.0288
k__Archaea;p__Crenarchaeota;c__Thaumarchaeota;o__Nitrososphaerales;f__Nitrososphaeraceae;g__Candidatus Nitrososphaera	.340*	0.034
k__Bacteria;p__Spirochaetes;c__[Leptospirae];o__[Leptospirales];f__Leptospiraceae;g__Turneriella	.333*	0.0385
k__Bacteria;p__Verrucomicrobia;c__[Spartobacteria];o__[Chthoniobacterales];f__[Chthoniobacteraceae];g__OR-59	.331*	0.0397
k__Bacteria;p__Chloroflexi;c__Anaerolineae;o__Caldilineales;f__Caldilineaceae;g__Litorilinea	.325*	0.0435
k__Bacteria;p__Chloroflexi;c__Anaerolineae;o__Anaerolineales;f__Anaerolinaceae;g__Anaerolinea	-.324*	0.0471
k__Archaea;p__Euryarchaeota;c__Methanomicrobia;o__Methanocellales;f__Methanocellaceae;g__Methanocella	-.323*	0.0478
k__Bacteria;p__Chloroflexi;c__Anaerolineae;o__Anaerolineales;f__Anaerolinaceae;g__SHD-231	.317*	0.0496
k__Bacteria;p__Proteobacteria;c__Alphaproteobacteria;o__Rhizobiales;f__Xanthobacteraceae;g__Labrys	-.320*	0.0499

* represent relevant on the level of 0.05. **represent relevant on the level of 0.01.
